# Cardiac Metastasis From Renal Cell Carcinoma

**DOI:** 10.7759/cureus.36439

**Published:** 2023-03-20

**Authors:** Bradley Casey, Amol Bahekar, Divyang Patel, Raviteja Guddeti, Selvaratnam Sinnapunayagam

**Affiliations:** 1 Internal Medicine, Cape Fear Valley Medical Center, Fayetteville, USA; 2 Cardiology, Cape Fear Valley Medical Center, Fayetteville, USA; 3 Cardiovascular Medicine, Creighton University School of Medicine, Omaha, USA

**Keywords:** lung metastasis, left atrial mass, lung cancer, left atrium, renal cell carcinoma (rcc)

## Abstract

While metastasis is common, it is unusual for renal cell carcinoma (RCC) to spread to the heart and even more so without involving the inferior vena cava (IVC). In fact, only a few cases have been reported where RCC has metastasized to the heart without IVC invasion. There have been only a few cases published that show RCC metastasis to the heart without invasion through the IVC. Here, we present an interesting case of a patient that was found to have RCC metastasis to the lungs that had a direct invasion to the left atrium.

## Introduction

In 2008, renal cell carcinoma (RCC) caused the highest number of deaths among all urologic cancers in Europe, with an estimated 44,570 fatalities [1]. Since cardiac tumors are rare and usually metastatic, only 0.8% of 266 cardiac neoplasms autopsied were primary tumors [2,3]. According to another study of 12,485 autopsies, primary and secondary heart tumors occur at rates of 0.06% and 1.23%, respectively, with metastasis most commonly occurring in the pericardium [2,4]. In addition to involving 2% to 3% of all cancers, this disease is highly aggressive, typically spreading to the lungs, liver, bones, and brain. [2,5]. Although metastasis to the heart is uncommon, it is even more rare for RCC to metastasize to the left atrium [2,5]. Here, we present an interesting case of RCC metastasis to the lung that infiltrated the left atrium.

## Case presentation

An 81-year-old African American male with past medical history (PMHx) of hypertension (HTN), tobacco abuse, pulmonary nodules, and RCC status post radical nephrectomy presented to the cardiology clinic by the request of his oncologist for a transesophageal echocardiogram (TEE). One year prior to this office visit, the patient was seen at a local hospital for hemoptysis. During that hospitalization, he had an esophagogastroduodenoscopy (EGD) that showed erosive esophagitis. Part of the hemoptysis workup in the emergency department was a computed tomography (CT) scan (we do not have access to these images), which showed a 5.8 cm mass in the right lung. The patient then proceeded with a CT-guided biopsy of the right lung mass, which showed metastatic RCC with papillary features. The patient was informed about the results, and he was told to follow up with his oncologist. Once the patient followed up with oncology, he was hesitant about treatment and wanted to discuss the treatment with his family. The patient followed up with his primary care provider for post-hospitalization follow-up, and a colonoscopy was ordered at the request of the patient at this time. The patient was lost to follow up with oncology, and after the colonoscopy, he came back to the oncologist after it showed a hyperplastic polyp in the distal sigmoid colon and tubular adenoma in the transverse colon. The patient was worried about these polyps due to his RCC. At this office visit one year later, the patient was open to treatment and was started on pembrolizumab (KEYTRUDA^®^) 200 mg in sodium chloride 0.9% 258 mL chemo IV piggyback (IVPB), 200 mg, intravenous.

**Figure 1:**
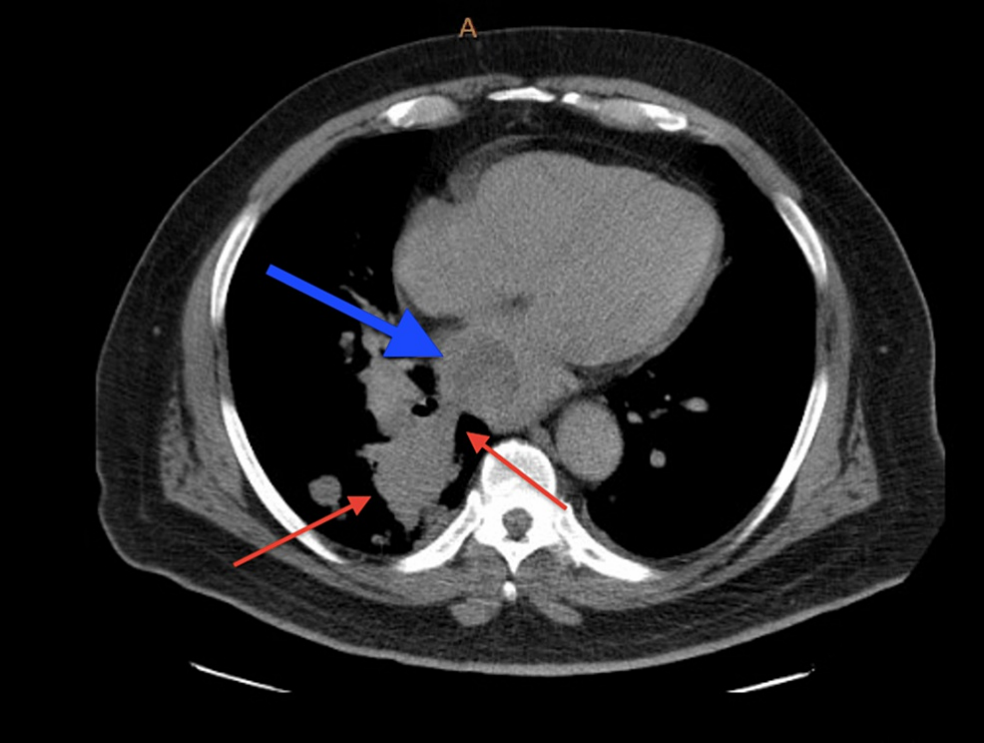
CT scan showing large right infrahilar mass consistent with malignancy (red arrows) with tumor invasion of the vasculature to the right lower lobe. Tumor invasion of the left atrium (blue arrow) of the heart versus left atrial thrombus.

**Figure 2:**
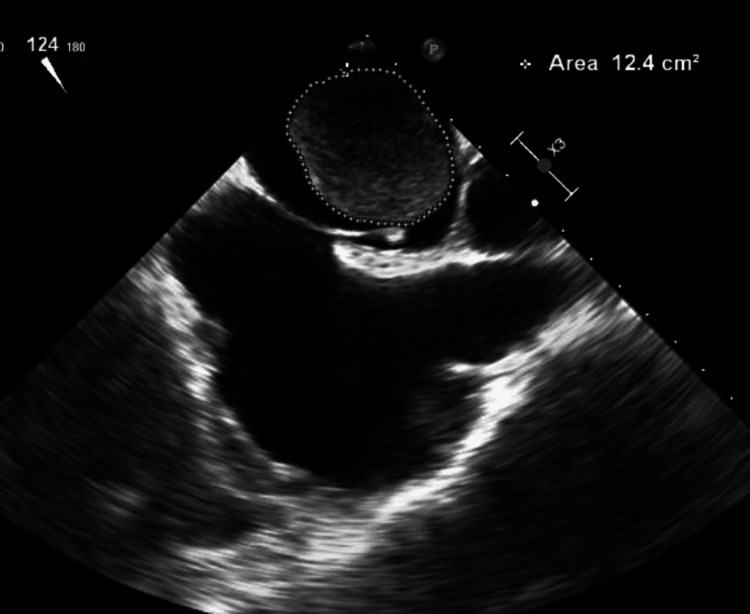
Area of the left atrial mass (12.4 cm square)

**Figure 3:**
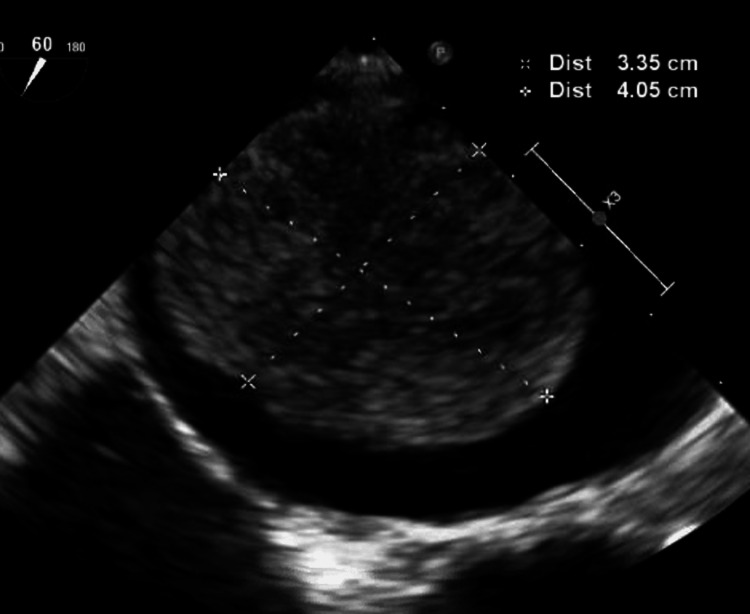
Dimensions of the left atrial mass (3.35 cm x 4.05 cm)

At this time, another CT scan of the chest was ordered (Figure 1), which showed a large right infrahilar mass and tumor invasion of the left atrium of the heart versus left atrial thrombus. The patient was then asked to follow up with cardiology for TEE. Then, TEE was performed, which showed an enlarged left atrium and a 3.35 cm x 4.05 cm left atrial mass with an area of 12.4 cm^2^ (Figures 2, 3 and Videos 1, 2). The patient was consistent in appointments, and he was offered surgical intervention for the left atrial mass, but he declined any surgical intervention. The patient is still continuing pembrolizumab treatment and has continued to follow up with oncology and cardiology.

**Video 1:** Transesophageal echocardiogram of the left atrial mass

**Video 2:** Transesophageal echocardiogram 3D reconstruction of the left atrial mass

## Discussion

About three-quarters of cardiac tumors are benign, nearly half are myxomas, and about 10% are lipomas [6]. Because RCC rarely metastasizes to the heart, its diagnosis is difficult, and patients are mostly asymptomatic [2]. This is likely why a majority of research on cardiac tumors is from post-mortem research. It is estimated that 20%-38% of patients with cardiac tumors suffer from HTN; other cardiac symptoms include syncope, dyspnea, chest pain, coughing, and peripheral edema [7]. Myocardial infarction or heart failure can result from cardiac tumors that compress or obstruct one or more coronary arteries [2].

There are three ways of tumor metastasis into the cardiac tissue: (1) direct mediastinal infiltration of heart tissue in cases of lung cancer; (2) metastatic formation by systemic tumors; (3) transvenous spreading from the vena cava inferior in cases of renal or hepatic tumors [6]. Typically, direct tumor thrombus extension into the inferior vena cava (IVC) causes cardiac involvement in RCC [8].

In every patient with cancer that includes distant metastases or thoracic involvement, the potential for cardiac metastasis should be taken into account [9]. Several physical examination findings, such as distant heart sounds, pericardial effusion, new murmurs from intracardiac masses, or a pericardial friction rub from pericarditis, may indicate the presence of cardiac metastases [9]. The most frequent abnormalities on an electrocardiogram are nonspecific ST-T-wave abnormalities and new atrial arrhythmias [9]. Even though most heart metastases are clinically quiet, cardiac metastases are rarely found during regular staging procedures [10]. Another factor is that motion artifacts in whole-body CT may prevent the detection of cardiac metastases [10]. The first imaging modality to identify any cardiac metastasis is echocardiography, which can also reveal details on the location, size, and mobility of heart masses [9]. The echocardiographic examination's sensitivity to detect cardiac metastasis was 75.9% [11].

For patients with metastatic RCC, molecularly targeted treatments have historically been the treatment of choice, but in the last two to three years, checkpoint inhibitor immunotherapy has become the standard of care [12]. According to the society for immunotherapy of cancer, as one of the new standards in the field, every patient should receive an anti-programmed death-1-based therapy as initial treatment [13]. Human immunoglobulin G4 monoclonal antibody, which binds to the programmed death-1 (PD-1) receptor, is the focus of current immunotherapy for metastatic RCC [12]. For patients with metastatic RCC, immunotherapy with these new antineoplastic-anti-programmed cell death receptor-1 monoclonal antibodies has become a crucial treatment option [12]. The effectiveness and safety of checkpoint inhibitors for patients with uncommon metastatic locations, such as intracardiac metastases, are currently unknown due to a lack of available evidence. Even with the novel antineoplastic-anti-programmed cell death receptor-1 monoclonal antibody, cardiac involvement without IVC involvement is extremely uncommon and presents a special therapeutic challenge [12].

## Conclusions

Here, we presented an interesting case of a patient that developed a left atrial mass from metastatic RCC of the lung. Only a few cases have been reported that show RCC of the heart without invasion from the IVC. This case shows the importance of imaging with a patient that has RCC metastasis to the lungs. Within one year, our patient's RCC had invaded his left atrium, and as clinicians, we need to keep this possibility in our differentials.
